# Prevalence and significance of *Mycoplasma genitalium* in women living with HIV in Denmark

**DOI:** 10.1186/s13104-017-2776-5

**Published:** 2017-09-07

**Authors:** Anne Marie Rosendahl Madsen, Kristina Thorsteinsson, Anne-Mette Lebech, Merete Storgaard, Terese L. Katzenstein, Frederikke F. Rönsholt, Isik Somuncu Johansen, Gitte Pedersen, Lars Noerregaard Nielsen, Aase Bengaard Andersen, Jørgen Skov Jensen

**Affiliations:** 1 Department of Clinical Microbiology, Odense University Hospital, J.B. Winsloews Vej 21, 2, 5000 Odense C, Denmark; 2 Department of Infectious Diseases, Copenhagen University Hospital, Hvidovre, Denmark; 3 Department of Infectious Diseases, Aarhus University Hospital, Aarhus, Denmark; 4 Department of Infectious Diseases, Copenhagen University Hospital, Rigshospitalet, Copenhagen, Denmark; 5 Department of Infectious Diseases, Odense University Hospital, Odense, Denmark; 6 Department of Infectious Diseases, Aalborg University Hospital, Aalborg, Denmark; 7 Department of Infectious Diseases, Hillerød Hospital, Hillerød, Denmark; 8 Statens Serum Institut, Copenhagen, Denmark

**Keywords:** *Mycoplasma genitalium*, HIV, Women living with HIV, Sexually transmitted diseases

## Abstract

**Objective:**

*Mycoplasma genitalium* (*M. genitalium*) is a sexually transmitted pathogen associated with urethritis, cervicitis, and pelvic inflammatory disease. Previous studies have shown a strong association between *M. genitalium* and HIV infection, therefore screening and treatment for *M. genitalium* has been suggested as part of HIV prevention strategies. The objective of this study was to determine the prevalence of *M. genitalium* in women living with HIV (WLWH) in Denmark, and to compare the result with data on symptoms from the lower abdomen, sexual habits and immune status. 234 women, recruited from Danish HIV centres as part of a larger observational study on aspects of living with HIV as a woman (the SHADE study), were included.

**Results:**

We tested cervical samples for *M. genitalium* by specific PCR. We found three samples positive (1.3%). The women were between 30 and 50 years old, all were of Asian origin, sexually active, and on antiretroviral treatment with supressed HIV RNA and CD4 count >350 cells/µL. None reported symptoms from the lower abdomen. The prevalence of *M. genitalium* infection in WLWH in Denmark is low, thus systematic screening for *M. genitalium* in this group does not seem relevant.

**Electronic supplementary material:**

The online version of this article (doi:10.1186/s13104-017-2776-5) contains supplementary material, which is available to authorized users.

## Introduction

*Mycoplasma genitalium* (*M. genitalium*) is a well-known sexually transmitted pathogen. It is an important cause of non-gonococcal urethritis in men, and in women it has been associated with cervicitis, urethritis and pelvic inflammatory disease [1]. Data from the last decade show a strong association between *M. genitalium* and HIV [2–4]. Women living with HIV (WLWH) infected with *M. genitalium* have been found to shed higher amounts of HIV in vaginal fluids, thereby possibly enhancing HIV transmission [5]. Infection with *M. genitalium* can lead to cervical inflammation, and it is possible that the infection also increases the risk of acquiring HIV infection, as is the case in other sexually transmitted diseases (STD) [6].

The prevalence of *M. genitalium* in women in the general population of high-income countries ranges from 1 to 4%, but is 10% or even higher among women attending STD clinics [7–9]. In the US, a survey of WLWH showed a 9.9% prevalence of *M. genitalium* infection [10]. Studies from Africa have documented a much higher prevalence in risk groups [11]. They also documented higher prevalence of *M. genitalium* infection among HIV positive women compared with HIV negative women, and it seems the infection persists longer in the HIV positive [12, 13]. One longitudinal study showed a twofold increased risk of being infected with HIV when *M. genitalium* positive [14].

Established risk factors for acquiring *M. genitalium* infection are young age, multiple sexual partners, smoking, black ethnicity, and possibly bacterial vaginosis [9, 15].

There are no data on *M. genitalium* infection in WLWH in Denmark or other parts of Scandinavia. We obtained cervical samples from WLWH participating in a larger observational study on aspects of living with HIV as a woman (the SHADE study). The aim of this study was to determine the prevalence of *M. genitalium* in WLWH in Denmark, and secondly to compare the result with data on symptoms from the lower abdomen, sexual habits and immune status.

## Main text

### Registries

*The Civil Registration System* (*CRS*) The CRS is a national registry of all Danish residents [16]. A 10-digit personal identification number (PIN) is assigned to everyone at birth or immigration. The PIN was used as a linkage to the Danish HIV Cohort Study (DHCS).

*Danish HIV Cohort Study* (*DHCS*) The DHCS is a prospective, observational, nationwide cohort study of all people living with HIV seen at the Danish HIV clinics since January 1995 [17]. From the DHCS we obtained data on baseline HIV characteristics.

### Methods

#### Study design

We tested cervical samples collected from 234 WLWH. Women were recruited from six Danish HIV centres as part of the SHADE cohort; study on HIV, cervical abnormalities and infections in women in Denmark. The SHADE cohort is a multicenter, prospective, observational cohort study of WLWH in Denmark attending regular outpatient care for their HIV infection. The study focuses on STD, contraception, sexual activity, human papillomavirus (HPV) infection, cervical cytological abnormalities, and other aspects of living with HIV as a woman. Results from the SHADE study have been published elsewhere [18].

Women were asked to participate if they had a known HIV-1 infection and were ≥18 years of age. Exclusion criteria were pregnancy and alcohol and/or drug abuse impeding adherence to the protocol. Of 1392 eligible women in the DHCS, 334 were included in the SHADE cohort. Women were enrolled between February 2011 and February 2012. The 234 WLWH included in the present study were those attending the 2 years follow-up of the SHADE study from February 2013–February 2014. At enrolment, women were tested for *Chlamydia trachomatis*, *Neisseria gonorrhoeae*, syphilis, and *herpes simplex* (HSV-1 and HSV-2). Gynaecological examinations were performed at entry and at 6, 12 and 24 months’ follow-up. An interview survey was performed to obtain background information. The questionnaire used has been described in detail elsewhere [18].

At entry, written and oral informed consent was obtained from all participants. The protocol has been approved by the Danish Data Protection Agency and by the Danish National Committee on Health Research Ethics.

#### Study population

The median age at inclusion in the study was 44 years (range 25–78 years). Treatment status was generally good, with 94% of the women on antiretroviral treatment (ART), and 86% on ART with an undetectable HIV RNA. CD4 count was 350 cells/µL or above in 80% of the cases. Symptoms from the lower abdomen were reported in 21% of the cases, most frequently vaginal discharge and abnormal menstrual bleeding. When asked about the number of lifetime sexual partners, 67% of the women reported less than 15 partners, 33% reported more than 15 partners, and of those, 18% had more than 25 lifetime sexual partners. When asked about sexual activity in the previous 6 months up to the follow up visit, 71% were sexually active. Furthermore, 56% were married or cohabitating and 32% reported to be single. Baseline characteristics of the WLWH are listed in Table 1.

**Table 1:** Baseline characteristics of study participants (n = 234)

Duration of HIV infection (years), median (IQR)	12.7 (7.5–17.8)
Age at inclusion (years), median (IQR)	44.4 (38.8–50.8)
Race, n (%)	
White	105 (45.1)
Asian	32 (13.7)
Black	94 (40.3)
Other	2 (0.9)
Missing	1
Place of HIV transmission, n (%)	
Denmark	84 (39.6)
Europe + US	16 (7.6)
Africa	86 (40.6)
Asia	26 (12.3)
Other	0 (0)
Missing	22
Mode of HIV transmission, n (%)	
Heterosexual	210 (92.5)
IDU	12 (5.3)
Other	5 (2.2)
Missing	7
CD4 count at inclusion (cells/μL), n (%)	
<200	11 (5.0)
200–350	33 (14.9)
>350	177 (80.1)
Missing	13
ART at inclusion, n (%)	
Yes	220 (94.0)
No	14 (6.0)
On ART with undetectable HIV RNA^a^, n (%)	
Yes	182 (85.5)
No	31 (14.6)
Missing	9
Lifetime sexual partners at inclusion, n (%)	
<4	58 (24.8)
5–9	57 (24.4)
10–14	40 (17.1)
15–25	36 (15.4)
26–40	13 (5.5)
>40	29 (12.4)
Does not wish to respond	1 (0.4)
Sexual activity in the past 6 months, n (%)	
Yes	165 (70.8)
No	68 (29.2)
Missing	1
Symptoms from the lower abdomen, n (%)	
Yes	50 (21.4)
No	184 (78.6)
Marital status	
Married	77 (41.2)
Cohabitating	27 (14.4)
Regular partner (not cohabitating)	23 (12.3)
Single	60 (32.1)
Missing	47
Smoking status at inclusion, n (%)	
Current smoker/ex-smoker	98 (41.9)
Never smoker	136 (58.1)

*ART* antiretroviral treatment, *IDU* intravenous drug use

^a^Undetectable = HIV RNA <40 copies/mL

#### Analyses

Samples were obtained by the treating physician performing a gynecological examination. Flocked swabs were collected in UTM transport medium, and *M. genitalium* was detected by TaqMan™ PCR amplifying a conserved region of the MgPa adhesin gene [19]. Positive results were confirmed by conventional PCR amplifying the 23S rRNA gene, and the resulting PCR products were subsequently sequenced to detect macrolide resistance mediating mutations [20]. The women who tested positive for *M. genitalium* were offered treatment with oral azithromycin for 5 days (500 mg day one, followed by 250 mg daily day 2–5). Per protocol treatment in case of macrolide resistance was oral moxifloxacin 400 mg daily for 7 days (Additional file 1).

#### Statistical analyses

Continuous variables were summarized as median and interquartile ranges (IQR) and compared using Wilcoxon rank sum test. Categorical variables were reported as counts and percentages and compared with Chi square test or Fisher’s exact test as appropriate. Further details on the statistical analyses on the SHADE cohort have been published previously [18].

## Results

Of the 234 women, three (1.3%) were PCR positive for *M. genitalium*. All carried a macrolide susceptible strain. The prevalence was too low to perform adjusted analyses aiming at predicting associated factors.

Of the WLWH in this study, 45% were white, 40% were black and 14% were Asian. Interestingly, all three *M. genitalium* positive women were from Asia (Thailand); thus, the prevalence among women from Asia was 10% (95% CI 2.0–27.0%) which was significantly higher than the 0% prevalence found in both white and black women (p = 0.01 and p = 0.02, respectively). Characteristics of the *M. genitalium* positive women are shown in Table 2.

**Table 2:** *Mycoplasma genitalium* positive women

*Mycoplasma genitalium* positive women	#1	#2	#3
Age group at inclusion (years)^a^	30–40	40–50	30–40
Race	Asian	Asian	Asian
Origin	Thailand	Thailand	Thailand
Place of HIV transmission	Asia	Asia	Asia
Mode of HIV transmission	IDU	Heterosexual	Heterosexual
CD4 (cells/µL)	>350	>350	>350
HIV RNA load	Undetectable	Undetectable	Undetectable
Smoking	Smoker	Non-smoker	Smoker
On ART	Yes	Yes	Yes
Sexually active	Yes	Yes	Yes
Positive for other STD^b^	No	No	No
Lifetime sexual partners	>40	<4	>40
Marital status	Unknown	Married	Single
Symptoms from the lower abdomen	None	None	None

*IDU* intravenous drug-use, *ART* antiretroviral treatment, *STD* sexually transmitted diseases

^a^Age is indicated as a 10-year range

^b^At inclusion

## Discussion

We found a relatively low prevalence of 1.3% of *M. genitalium* infections in this population. The result is comparable to the prevalence of 1–4% reported in general populations in high-income countries [7–9], but much lower than in studies of African and American WLWH, where the prevalence generally has been much higher [11, 21]. Interestingly, all *M. genitalium* infected women were from Thailand. Although numbers were small, all three were infected with macrolide susceptible strains of *M. genitalium*, which may be surprising as the level of macrolide resistance in Thailand is expected to be high, although no precise figures exist. If the women were infected in Denmark, we would expect nearly half to carry resistance mediating mutations [20]. The low prevalence of *M. genitalium* could be explained by several factors. The median age is relatively high in the present study compared to other studies of both WLWH and general populations. Furthermore, the women in this population seemed to be less sexually active than WLWH in other study populations. The majority of the women were in a relationship or had a regular partner. A recent study from France showed a similar low prevalence of *M. genitalium* (3.8%) among WLWH [22]. The study population was comparable to the present study population with respect to the median age (41.3 and 44 years respectively), race (42 and 55% non-white respectively), and sexual activity. Many of the studies carried out in Africa included women in high-risk populations.

## Conclusions

The prevalence of *M. genitalium* was relatively low in this study population. The result is comparable to the prevalence reported in the general populations in high-income countries. Screening for *M. genitalium* as part of HIV prevention strategies does not seem relevant in this setting.

## Limitations

With only three positive results, there was no basis for a statistical analysis of risk factors associated with *M. genitalium* infection. The women who participated in this study were generally older and less sexually active than the women in most other studies of HIV and *M. genitalium*, and most of them were successfully treated with fully supressed HIV RNA.

## Additional file

**Additional file 1.** Dataset with the results of *M. genitalium* PCR on the cervical samples.

## Abbreviations

*M. genitalium*: *Mycoplasma genitalium*
HIV: human immunodeficiency virus
WLWH: women living with HIV
PCR: polymerase chain reaction
ART: antiretroviral treatment
STD: sexually transmitted disease
SHADE cohort: study on HIV, cervical abnormalities and infections in women in Denmark
HPV: human papillomavirus
HSV: herpes simplex virus
CRS: Civil Registration System
DHCS: Danish HIV Cohort Study
UTM: universal transport medium
CI: confidence interval

## References

[1] Taylor-Robinson D, Jensen JS (2011). *Mycoplasma genitalium*: from Chrysalis to multicolored butterfly. Clin Microbiol Rev.

[2] Mavedzenge SN, Weiss HA (2009). Association of *Mycoplasma genitalium* and HIV infection: a systematic review and meta-analysis. Aids.

[3] Mavedzenge SN, Muller EE, Lewis DA, Chipato T, Morrison CS, Weiss HA (2015). *Mycoplasma genitalium* is associated with increased genital HIV type 1 RNA in Zimbabwean women. J Infect Dis.

[4] Vandepitte J, Weiss HA, Bukenya J, Kyakuwa N, Muller E, Buve A, Van der Stuyft P, Hayes RJ, Grosskurth H (2014). Association between *Mycoplasma genitalium* infection and HIV acquisition among female sex workers in Uganda: evidence from a nested case–control study. Sex Transm Infect.

[5] Manhart LE, Mostad SB, Baeten JM, Astete SG, Mandaliya K, Totten PA (2008). High *Mycoplasma genitalium* organism burden is associated with shedding of HIV-1 DNA from the cervix. J Infect Dis.

[6] Fleming DT, Wasserheit JN (1999). From epidemiological synergy to public health policy and practice: the contribution of other sexually transmitted diseases to sexual transmission of HIV infection. Sex Transm Infect.

[7] Andersen B, Sokolowski I, Ostergaard L, Kjolseth Moller J, Olesen F, Jensen JS (2007). *Mycoplasma genitalium*: prevalence and behavioural risk factors in the general population. Sex Transm Infect.

[8] Weinstein SA, Stiles BG (2011). A review of the epidemiology, diagnosis and evidence-based management of *Mycoplasma genitalium*. Sex Health.

[9] Cazanave C, Manhart LE, Bebear C (2012). *Mycoplasma genitalium*, an emerging sexually transmitted pathogen. Med Mal Infect.

[10] Gatski M, Martin DH, Theall K, Amedee A, Clark RA, Dumestre J, Chhabra P, Schmidt N, Kissinger P (2011). *Mycoplasma genitalium* infection among HIV-positive women: prevalence, risk factors and association with vaginal shedding. Int J STD AIDS.

[11] Vandepitte J, Muller E, Bukenya J, Nakubulwa S, Kyakuwa N, Buve A, Weiss H, Hayes R, Grosskurth H (2012). Prevalence and correlates of *Mycoplasma genitalium* infection among female sex workers in Kampala, Uganda. J Infect Dis.

[12] Vandepitte J, Weiss HA, Kyakuwa N, Nakubulwa S, Muller E, Buve A, Van der Stuyft P, Hayes R, Grosskurth H (2013). Natural history of *Mycoplasma genitalium* infection in a cohort of female sex workers in Kampala, Uganda. Sex Transm Dis.

[13] Cohen CR, Nosek M, Meier A, Astete SG, Iverson-Cabral S, Mugo NR, Totten PA (2007). *Mycoplasma genitalium* infection and persistence in a cohort of female sex workers in Nairobi, Kenya. Sex Transm Dis.

[14] Mavedzenge SN, Van Der Pol B, Weiss HA, Kwok C, Mambo F, Chipato T, Van der Straten A, Salata R, Morrison C (2012). The association between *Mycoplasma genitalium* and HIV-1 acquisition in African women. Aids..

[15] Oakeshott P, Aghaizu A, Hay P, Reid F, Kerry S, Atherton H, Simms I, Taylor-Robinson D, Dohn B, Jensen JS (2010). Is *Mycoplasma genitalium* in women the “New Chlamydia?” A community-based prospective cohort study. Clin Infect Dis.

[16] Pedersen CB (2011). The Danish Civil Registration System. Scand J Public Health.

[17] Obel N, Engsig FN, Rasmussen LD, Larsen MV, Omland LH, Sorensen HT (2009). Cohort profile: the Danish HIV cohort study. Int J Epidemiol.

[18] Thorsteinsson K, Ladelund S, Storgaard M, Ronsholt FF, Johansen IS, Pedersen G, Nielsen LN, Bonde J, Westh H, Obel N (2016). Sexually transmitted infections and use of contraceptives in women living with HIV in Denmark—the SHADE cohort. BMC Infect Dis.

[19] Jensen JS, Bjornelius E, Dohn B, Lidbrink P (2004). Use of TaqMan 5′ nuclease real-time PCR for quantitative detection of *Mycoplasma genitalium* DNA in males with and without urethritis who were attendees at a sexually transmitted disease clinic. J Clin Microbiol.

[20] Salado-Rasmussen K, Jensen JS (2014). *Mycoplasma genitalium* testing pattern and macrolide resistance: a Danish nationwide retrospective survey. Clin Infect Dis.

[21] Gomih-Alakija A, Ting J, Mugo N, Kwatampora J, Getman D, Chitwa M, Patel S, Gokhale M, Kimani J, Behets FS, Smith JS (2014). Clinical characteristics associated with *Mycoplasma genitalium* among female sex workers in Nairobi, Kenya. J Clin Microbiol.

[22] Cazanave C, Lawson-Ayayi S, Hessamfar M, Neau D, Dupon M, Morlat P, Dabis F, de Barbeyrac B, Bebear C, Pereyre S (2013). Prevalence of *Mycoplasma genitalium* among HIV-infected women, Agence Nationale de Recherches sur le SIDA et les hepatites virales CO3 Aquitaine Cohort, France. Sex Transm Dis.

